# Operative time, recurrence, and complications throughout the initial learning curve in transforaminal endoscopic lumbar discectomy

**DOI:** 10.1007/s00701-026-06770-1

**Published:** 2026-01-21

**Authors:** Michelle D. Poelman, Annegien Boeykens, Biswadjiet S. Harhangi, Marc L. Schröder, Victor E. Staartjes

**Affiliations:** 1 Department of Neurosurgery, Park Medical Center, Rotterdam, The Netherlands; 2 Department of Neurosurgery, Erasmus University Medical Center, Rotterdam, The Netherlands; 3 Machine Intelligence in Clinical Neuroscience & Microsurgical Neuroanatomy (MICN) Laboratory, Department of Neurosurgery, Clinical Neuroscience Center, University Hospital Zurich, University of Zurich, Zurich, Switzerland; 4 Capio Spine Center Stockholm, Löwenströmska Hospital, Upplands-Väsby, Sweden; 5 Department of Clinical Neuroscience, Karolinska Institutet, Stockholm, Sweden

**Keywords:** Discectomy, Herniated disc, Learning curve, Endoscopic, Recurrence, Complication

## Abstract

**Background:**

Transforaminal Endoscopic Lumbar Discectomy (TELD) is increasing in popularity as a minimally invasive technique for treating lumbar disc herniation (LDH). However, TELD presents technical challenges that may result in a flat learning curve. This study analysed the first patients treated by a single senior neurosurgeon transitioning from tubular microdiscectomy, focussing on the initial learning curve for operative time, recurrence, and complications.

**Methods:**

A retrospective study was conducted using data from a consecutive cohort of the first 213 patients operated for LDH by TELD. We collected basic demographic data and recorded all complications, recurrences, and operative time, among other clinical outcome measures. For analytical purposes (trend testing), the learning curve was divided into four quarters of each approx. 50 patients.

**Results:**

The cohort included 101 (47.4%) females and 112 (52.6%) males, with a mean age of 44.2 ± 11.8 years. An initial steep decrease in operative time after 50 cases performed was observed, decreasing by 21.9 ± 27.7 min (*p* < 0.001), with operative time showing no further change after those initial 50 cases. Residual LDH was seen in 2 (0.9%) patients. Seventeen (8.0%) patients experienced recurrence of LDH. No statistically significant trend in recurrence rate between quarters was observed (*p* = 0.99). Complications were experienced by 11 (5.2%) patients, without a significant trend (*p* = 0.50).

**Conclusions:**

This study demonstrates a clear and steep learning curve for TELD, as shown by a significant decrease in operative time that stabilized after approximately 50 cases. This rapid improvement shows growing familiarity with the technically demanding procedure. In our experience, the initial step of the docking process remains the most challenging aspect, largely due to patient-specific variations. Understanding the initial learning curve is essential for training and surgical planning when transitioning to endoscopic techniques.

## Introduction

Lumbar disc herniation (LDH) is a common spinal condition, most frequently occurring at the L4-L5 or L5-S1 level [3]. While a conservative approach is most often effective, patients with persistent or intractable leg pain or neurological deficits may require surgical intervention. Although open microdiscectomy (OM) remains the gold standard for surgical treatment of LDH, Transforaminal Endoscopic Lumbar Discectomy (TELD) is becoming more popular as a minimally invasive alternative for treating LDH [4, 7]. Recent studies have shown that TELD provides comparable outcomes to OM in terms of leg pain or functional outcomes [6, 14], and may be more cost effective from a societal perspective [8].

TELD has several benefits, including the ability to perform surgery under local anaesthesia, a small skin incision, and provides endoscopic visualization [14]. Its lateral transforaminal approach avoids dissection of paravertebral muscles and preserves original bony anatomy as well as other surrounding tissue [10, 14]. TELD has been reported to offer shorter hospital stays, less surgical trauma, faster recovery, and improved relief of pain, sensory, and motor symptoms [1, 10]. However, differences between OM and TELD are often small and may lack clinical relevance [1, 9].

For many experienced surgeons, transitioning from microsurgical techniques to TELD is a time-intensive challenge. While there are some reports in the literature on the initial learning curve in TELD [8, 16, 18, 19], the learning curve of neurosurgeons with great experience in microdiscectomy transitioning to endoscopy at a later career stage is of particular interest. Our previous research examined the learning curve progression of a single experienced surgeon performing tubular Microdiscectomy (tMD), focusing on operative times and recurrence rates of LDH [17]. However, detailed investigation of the initial development of operative time and recurrence-, and complication rates throughout the initial learning curve in TELD have not been studied in much detail. This study aims to investigate this knowledge gap.

In this study, we analysed a consecutive series of the first patients treated by a single senior neurosurgeon after having completed > 9000 microtubular discectomies, with a particular focus on the initial learning curve for operative time, recurrence, and complications.

## Methods

### Study design

This study was conducted at a single high-volume short-stay spine center in the Netherlands. Outcomes of patients undergoing TELD for LDH were retrospectively collected in a consecutive cohort of the first patients treated by a single senior neurosurgeon. The surgeon (age 61) had no prior experience in endoscopic lumbar surgery, but had performed over 9,000 tMDs before the study period. Before performing TELD on patients, the surgeon M.S completed two cadaver courses over a period of two months. During the initial learning phase, an experienced endoscopic surgeon (B.H.) provided supervision. To evaluate the progression of the learning curve, the learning process was divided into 4 quarters, each containing approximately 50 patients.

### Study population

Patient data was collected of patients operated from December 2021 to December 2024, representing the first three years of the learning curve. Patients had undergone TELD in an outpatient setting. Due to the setting of the clinic, patients were not considered for surgery if they had an American Society of Anesthesiologists (ASA) score of more than 2, were aged > 80 years old, had a body mass index (BMI) of > 33 and if they were using anticoagulants (except for Acetylsalicylic acid).

### Surgical procedure

The surgical procedure was performed by the senior author (M.S.) who has extensive experience in tMD [17]. For a more detailed description of the surgical technique of TELD, we refer to a technical paper of Harhangi et al. [5]. The surgical instrumentation used for TELD remained consistent during the study period. All patients were positioned prone on the table on a Wilson frame. The procedure was performed under local anaesthesia with light sedation.

In summary, the entry point was verified under fluoroscopic guidance by placing a needle reaching the superior articular process. The facet joint was anesthetized with lidocaine and a needle was introduced. After that, the position of the needle was checked under fluoroscopy and a guidewire was inserted. Dilation was performed using conical rods. Drills of increasing size were subsequently introduced through the cannula to enlarge the neuroforamen. Then, the guidewire was replaced by a sharp and then a blunt TomShidi (MaxMorespine, Hoogland Spine Products GmbH, Germany) needle to perforate the superior articular process and enter the epidural space. The foramen was widened to 8–9 mm by further drilling of the lateral part of the superior articular process in an anticlockwise direction, thereby minimizing muscle damage. Finally, a working channel was introduced and the endoscope was placed.

To remove disc fragments, a rongeur was used under direct endoscopic visualization and using continuous irrigation with 0.9% saline. Adequate decompression was achieved when pulsations of the nerve root were clearly visible and the amount of removed disk material corresponded with the preoperative MRI findings. At this point, once pulsation was achieved, no further discectomy was performed. Irrigation fluid was pushed out with a dilator, the endoscope and working channel were removed, and the skin was closed with subcutaneous sutures.

### Outcome measures

We collected basic demographic data, including age, gender, BMI, smoking status, ASA scores, and level and side of LDH. We also recorded whether patients had already had previous LDH at the same level and side. Operative data included operative time, as well as peri- and postoperative complications.

The primary outcome measure was operative time in minutes and recurrence rate. A true recurrence was defined as a return of symptoms after an episode of pain relief, with MRI-confirmed LDH at the same level and the same side [17].

We also captured patients with persistent symptoms after TELD, sometimes requiring re-discectomy using tMD. This was classified as having a residual LDH rather than a true recurrence.

### Statistical analysis

Continuous data were reported as means and standard deviations, and categorical data as numbers and percentages. For analytical purposes, the initial experience was divided into quarters of approximately 50 patients each. This cut-off provided sufficiently granular subgrouping while preserving statistical power within each quarter to perform trend analyses. To visualize the learning curve on the one hand, boxplots and bar plots were used, demonstrating each quarter. On the other hand we also applied a moving average technique to plot the density of events over time. In addition, a cumulative sum (CUSUM) analysis was performed, calculating the cumulative difference between the operative time per case and the overall mean. The CUSUM plot highlights performance over time, with the peak of the CUSUM curve indicating the end of the initial learning phase. To assess the trend in operative time between quarters, a 2-tailed Jonckheere-Terpstra test was performed based on 10,000 permutations. The 2-tailed Cochrane-Armitage test was applied to test for trends in dichotomous variables (recurrence rate, complication rate). All analyses were carried out using R software version 4.5.0 (R Core Team, 2024) [13]. A 2-tailed p ≤ 0.05 was considered statistically significant.

## Results

### Patient characteristics

Between December 2021 and December 2024, a total of 213 patients underwent TELD performed by a single surgeon. The cohort included 101 (47.4%) females and 112 (52.6%) males, with a mean age of 44.2 ± 11.8 years. Detailed patient demographics are provided in Table 1.

**Table 1:** Baseline characteristics

Characteristic	Value
Number of patients, n (%)	213 (100)
Age in years, mean	44.2 ± 11.8
Female sex, n (%)	101 (47.4)
BMI in kg/m2, mean ± SD	25.3 ± 3.2
Smoker status, n (%)	
Active smoker	48 (23)
Ex-smoker	3 (1)
Non-smoker	162 (76)
ASA score, n (%)	
I	92 (43)
II	119 (56)
III	2 (1)
Herniation level, *n* (%)	
L4-L5	90 (42)
L5-S1	123 (58)
Side of herniation, *n* (%)	
Left	118 (55)
Right	95 (45)
Prior spine surgery, n (%)	22 (10)
Prior LDH at same level and side, n (%)	8 (4)

*SD* standard deviation

### Operative time

**Fig. 1:**
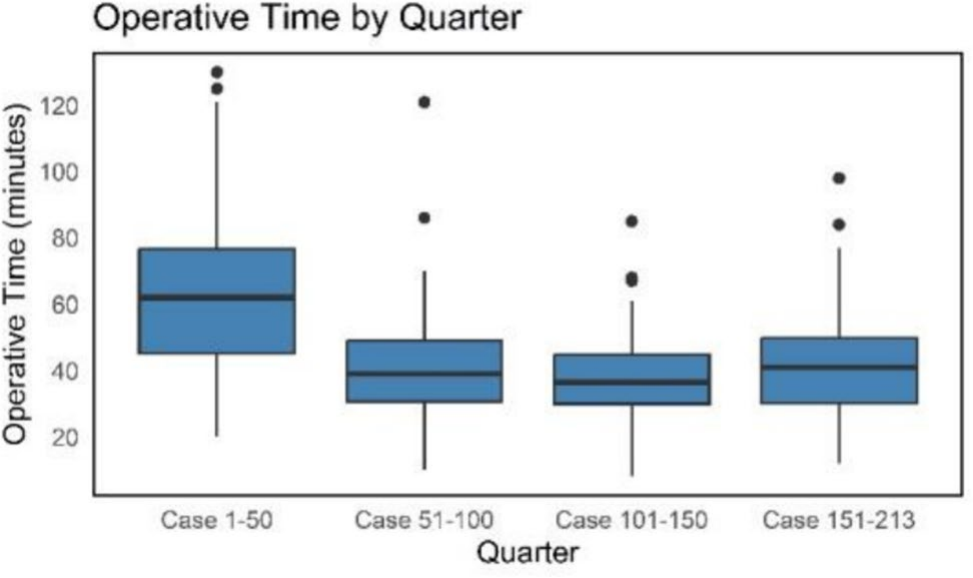
Operative time by quarter. Boxplot shows operative time in minutes per quarter

The Jonckheere-Terpstra test revealed a statistically significant decrease in operative time between quarters (JT = 6104.5, *p* < 0.001, Fig. 1), suggesting presence of a significant learning curve.

**Fig. 2:**
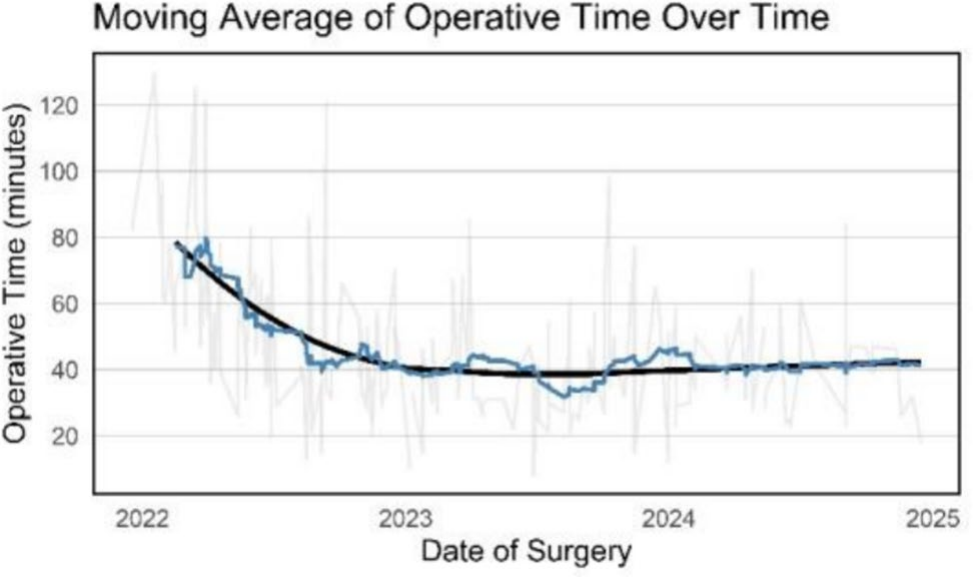
Moving average of operative time over time. Moving average graph of operative time over time. Each point on the blue curve represents the mean operative time over 20 consecutive surgeries (current and previous 19 cases). Raw data is shown in light grey and a LOESS curve in black highlights overall trends

To visualize trends in operative time, a 20-case moving average was computed (a sliding window of k = 20 consecutive surgeries) (Fig. 2). After the first quarter, the mean operative time decreased by 21.9 ± 27.7 min up to the second quarter. Thereafter, no meaningful changes can be appreciated. The last three quarters remained stable at 37.5 ± 15.5 min.

**Fig. 3:**
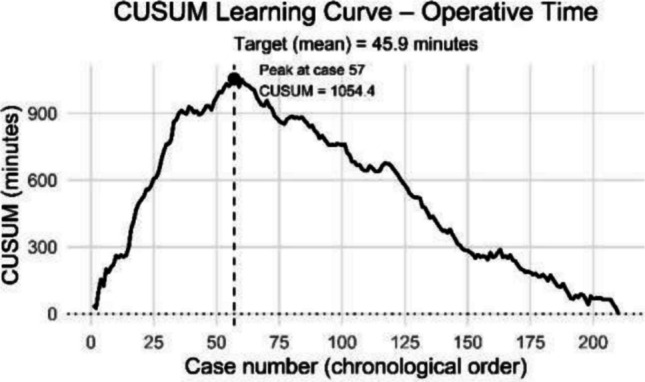
CUSUM analysis of operative time. The cumulative sum highlights the peak at case 57, marking the end of the initial learning phase. Baseline (dotted line) represents mean operative time

CUSUM analysis confirmed these findings (Fig. 3). The CUSUM curve peaked at case 57, after which operative time declined, indicating the end of the initial learning phase.

### Recurrence rate

True recurrence was seen in 17 patients (8.0%). The median time to recurrence was 70 days (IQR: 52–266). The Cochran-Armitage test showed no statistically significant trend in recurrence rate between quarters (Z = 0.012, *p* = 0.99), indicating that the recurrence rate did not significantly change as surgical experience increased. Residual LDH was seen in 2 (0.9%) patients.

### Complication rate

Eleven patients experienced complications, which gives a complication rate of 5.2%, including six surgeries (2.8%) requiring conversion to tMD. Other complications are listed in Table 2. The Cochran-Armitage test showed no statistically significant trend in complication rate between quarters (Z = −0.676, *p* = 0.50), suggesting that the complication rate did not significantly change over time.

**Table 2:** Summary of recurrence and complication rate

Characteristic	Value
Recurrence rate, n (%)	17 (8.0)
Residual LDH, n (%)	2 (0.9)
Time to recurrence, days, median (IQR*)	70 (214)
Complications, n (%)	
Conversion to tMD	6 (2.8)
Dural tear	3 (1.4)
Minor iatrogenic nerve root lesion**	2 (0.9)
Vasovagal response with severe bradycardia	1 (0.5)

*IQR interquartile range

**No paresis nor neurological deficits

## Discussion

In this study, we evaluated the learning curve for TELD of a senior surgeon with extensive experience transitioning from tMD. Operative time significantly decreased after 50 cases, and stabilized afterwards, highlighting a clear learning curve during the initial phase. Recurrence rates as well as complication rates showed no statistically significant trend, suggesting that these parameters are not influenced by growing experience.

Compared to our previous analysis on the learning curve in tMD [17], operative time decreased gradually across 1,240 cases, with an average reduction of 2.3 min per 310 cases. However, this was an analysis of a cohort of patients operated long after the initial tMD learning curve. This present study on TELD revealed a steeper learning curve: operative time decreased by an average of 21.9 min after the first 50 cases performed. After the first 50 patients, the surgeon becomes more comfortable with the complex docking part of the procedure (finding the correct trajectory). Anecdotally, though, even after achieving a notably faster operating time after the first 50 patients, we continued to experience the first part of the procedure (trajectory, docking) as highly demanding. Even though this is not reflected in the stabilization of the rest of the learning curve, we still find this to be by far the most challenging part of the procedure. As an example, technically speaking, an optimal trajectory should be achieved with an entry point that is, as recommended, 8 to 13 cm from the midline, depending on the level of the herniation [2, 5]. However, in reality, the measures do not take the variability of the patient into account: varying BMI’s, patient height, hypertrophy of the facet joints. These variations mean that there is no ‘one size fits all’ approach to finding the correct trajectory, and in practicality this often requires prolonged operative time and radiation exposure.

Navigation will ameliorate the docking process, taking into account the complexity of the first part of the procedure. Although its initial use may prolong operative time when starting this new technique, it has the potential to shorten and facilitate the docking process and duration of radiation exposure.

While TELD may be cost-efficient from a societal perspective [8], individual institutions may face higher costs during the initial learning curve because of higher disposable costs, longer operative times, and required technological investments. These factors should be considered when implementing TELD in new centers.

During the initial phase of the learning curve, it is highly advised to only select patients with a clear lateral recess location of the herniation, not migrated cranially or caudally, and not too far dorsal in the canal [11]. It is also recommended initially to select patients with a herniation located at the level L4-5, because the level L5-S1 is technically more challenging [12]. As more experience is gained, indications can be expanded to more challenging positions or sizes of the herniations.

Our anecdotal experience and findings highlight the technical demands of TELD in the initial learning curve. These results are not unexpected, as, unlike tMD where skills largely overlap with those required for open microscopic surgery, TELD requires unique technical and anatomical adaption by a shift towards endoscopic approach.

Differences in recurrence rate showed no statistically significance, suggesting the absence of a learning curve effect. However, the second quarter showed a notable decrease. This observation is likely due to chance. Still, we experienced modifications in the surgical strategy as familiarity with the technique increased. Specifically, the surgeon adopted a novel mindset about what constitutes adequate neural decompression, prioritizing the reestablishment of cerebrospinal fluid (CSF) pulsation rather than removing maximum disc tissue. Moreover, the use of awake surgery allowed for immediate patient’s feedback and confirmation of symptom relief. These changes may contribute to varying recurrence rates.

It is well known that most of the complications usually occur within the first phase of a learning curve [15, 16]. In our cohort, we found no statistically significant difference in complication rates. However, we did observe a slight increase across this study period (Table 3). However, these numbers are small and not statistically significant. This observed effect could be due to random variation. A larger sample size is needed to explore whether this trend holds.

**Table 3:** Complication and recurrence rate across quarters

Quarter	Complication rate per quarter, *n* (%)	Recurrence rate per quarter, *n* (%)
Case 1–50	2 (4.0)	6 (12.0)
Case 51–100	2 (4.0)	1 (2.0)
Case 101–150	3 (6)	4 (8.0)
Case 151–213	4 (6.3)	6 (9.5)

### Limitations

This study has several limitations. Because of its retrospective design, it is impossible to control for all variables. In addition, this study was done in a single-center setting by a single surgeon. This may limit the generalizability of the findings to other settings or surgeons with different experiences. Radiation exposure per case was not recorded in this series, preventing comparison to microdiscectomy and assessment of radiation trends during the learning curve.

Obese- (BMI > 33), and high-risk patients (ASA-score > 2) were not considered for surgery in this outpatient surgery setting. This may have influenced the absolute operative time, but should have no effect on the learning curve.

Furthermore, operative time was focused on to assess learning curve progression. It may not fully capture improvements such as decision-making and surgical confidence. Besides recurrence rate, other functional outcomes such as pain relief or quality of life were not evaluated and could have added further insight into the surgeon’s progression.

While recurrence rate was carefully defined and assessed, follow-up duration was limited, and some late recurrences may not have been captured.

Despite these limitations, this study provides valuable insight into the initial learning curve of TELD by an experienced surgeon.

## Conclusion

This study demonstrates a clear and steep learning curve for TELD, as shown by a significant decrease in operative time that stabilized after approximately 50 cases. This rapid improvement shows growing familiarity with the technically demanding procedure. Notably, the initial step of the docking process remains the most challenging aspect, largely due to patient-specific variations, where no ‘one size fits all’ approach can be used. Understanding the initial learning curve is essential for training and surgical planning when transitioning to endoscopic techniques.

## Data Availability

Data is available from the corresponding author upon reasonable request.

## Abbreviations

ASA: American Society of Anesthesiologists
BMI: Body Mass Index
CSF: Cerebrospinal fluid
CUSUM: Cumulative sum
IQR: Interquartile Range
LDH: Lumbar Disc Herniation
TELD: Transforaminal endoscopic lumbar discectomy
tMD: tubular Microdiscectomy
OM: Open Microdiscectomy
